# A practical agenda for incorporating trust into pandemic preparedness and response

**DOI:** 10.2471/BLT.23.289979

**Published:** 2024-04-30

**Authors:** Thomas J Bollyky, Michael Bang Petersen

**Affiliations:** a Council on Foreign Relations, 1777 F Street, NW, Washington, DC 20006, United States of America.; b Department of Political Science, Aarhus University, Aarhus, Denmark.

## Abstract

Despite widespread acknowledgement that trust is important in a pandemic, few concrete proposals exist on how to incorporate trust into preparing for the next health crisis. One reason is that building trust is rightly perceived as slow and challenging. Although trust in public institutions and one another is essential in preparing for a pandemic, countries should plan for the possibility that efforts to instil or restore trust may fail. Incorporating trust into pandemic preparedness means acknowledging that polarization, partisanship and misinformation may persist and engaging with communities as they currently are, not as we would wish them to be. This paper presents a practical policy agenda for incorporating mistrust as a risk factor in pandemic preparedness and response planning. We propose two sets of evidence-based strategies: (i) strategies for ensuring the trust that already exists in a community is sustained during a crisis, such as mitigating pandemic fatigue by health interventions and honest and transparent sense-making communication; and (ii) strategies for promoting cooperation in communities where people mistrust their governments and neighbours, sometimes for legitimate, historical reasons. Where there is mistrust, pandemic preparedness and responses must rely less on coercion and more on tailoring local policies and building partnerships with community institutions and leaders to help people overcome difficulties they encounter in cooperating with public health guidance. The regular monitoring of interpersonal and government trust at national and local levels is a way of enabling this context-specific pandemic preparedness and response planning.

## Résumé

Bien qu'il soit largement admis que la confiance est un facteur crucial lors d'une pandémie, peu de propositions concrètes ont été formulées quant aux modalités de son intégration dans la préparation aux prochaines crises sanitaires. L'une des raisons tient au fait qu'établir la confiance est considéré, à juste titre, comme un processus lent et complexe. La confiance mutuelle et celle placée dans les institutions publiques est essentielle dans la préparation aux pandémies, les pays devraient donc tenir compte de la possibilité que leurs efforts pour instaurer ou restaurer cette confiance échouent. Intégrer ce facteur dans la préparation aux pandémies signifie reconnaître que la polarisation, la partialité et la désinformation sont susceptibles de persister. Cela signifie aussi travailler avec les communautés telles qu'elles sont actuellement, et non telles que nous souhaiterions qu'elles soient. Le présent document dévoile un programme politique concret visant à inclure la méfiance comme facteur de risque dans la planification des mesures de préparation et de riposte aux pandémies. Nous suggérons deux types de stratégies fondées sur des données factuelles: (i) des stratégies visant à préserver la confiance préexistante au sein d'une communauté durant une crise, notamment en luttant contre la lassitude face aux pandémies par le biais d'interventions de santé et d'une communication honnête, transparente et sensée; mais aussi (ii) des stratégies qui favorisent la coopération dans les communautés dont les membres se méfient de leur gouvernement et de leurs voisins, parfois pour des raisons historiques légitimes. Lorsque des doutes subsistent, les mesures de préparation et de riposte aux pandémies doivent éviter de recourir à la contrainte et privilégier des politiques locales adaptées ainsi que des partenariats avec les responsables et les institutions de la communauté, afin d'aider les gens à surmonter les difficultés qu'ils rencontrent vis-à-vis des directives de santé publique. Un suivi régulier de la confiance envers les autres et les autorités à l'échelle régionale et nationale permet de planifier une préparation et une riposte spécifiques face aux pandémies.

## Resumen

A pesar del reconocimiento generalizado de que la confianza es importante en una pandemia, existen pocas propuestas concretas sobre cómo incorporarla a la preparación para la próxima crisis sanitaria. Uno de los motivos es que generar confianza se percibe, con razón, como algo lento y difícil. Aunque la confianza en las instituciones públicas y en los demás es esencial en la preparación para una pandemia, los países deben prever la posibilidad de que fracasen los esfuerzos por infundir o restablecer la confianza. Incorporar la confianza a la preparación ante una pandemia significa reconocer que la polarización, el partidismo y la desinformación pueden persistir y comprometerse con las comunidades tal y como son actualmente, no como desearíamos que fueran. Este documento presenta una agenda política práctica para incorporar la desconfianza como factor de riesgo en la planificación de la preparación y respuesta ante una pandemia. Proponemos dos conjuntos de estrategias basadas en la evidencia: (i) estrategias para garantizar que la confianza que ya existe en una comunidad se mantenga durante una crisis, como mitigar la fatiga pandémica mediante intervenciones sanitarias y una comunicación honesta y transparente que haga entrar en razón; y (ii) estrategias para promover la cooperación en comunidades donde las personas desconfían de sus gobiernos y vecinos, a veces por razones legítimas e históricas. Cuando hay desconfianza, la preparación y las respuestas ante una pandemia se deben basar menos en la coerción y más en la adaptación de las políticas locales y la creación de asociaciones con las instituciones y los líderes de la comunidad para ayudar a las personas a superar las dificultades que encuentran en la cooperación con las orientaciones de salud pública. El seguimiento periódico de la confianza interpersonal y gubernamental a nivel nacional y local es una forma de hacer posible esta planificación de la preparación y respuesta ante una pandemia específica para cada contexto.

## ملخص

على الرغم من الاعتراف على نطاق واسع بأن الثقة هامة في حالة الأوبئة، إلا أن هناك القليل من المقترحات الملموسة حول كيفية تعزيز الثقة في الاستعداد للأزمة الصحية المحتملة. أحد الأسباب وراء ذلك هو أن بناء الثقة يُنظر إليه على نحو ملائم باعتباره أمراً بطيئاً وشاقًا. وعلى الرغم من أن الثقة في المؤسسات العامة، وفي بعضها البعض، ضرورية في سبيل الاستعداد لمواجهة الوباء، يجب على الدول أن تحتاط لاحتمال فشل الجهود الرامية إلى غرس الثقة أو استعادتها. إن تعزيز الثقة في الاستعداد لمواجهة الأوبئة يعني الاعتراف بأن الاستقطاب، والتحزب، والمعلومات المضللة، قد تستمر جميعها، وأن التعامل مع المجتمعات كما هي حاليًا، وليس كما نتمنى لها أن تكون. تقدم هذه الورقة أجندة سياسية عملية لتعزيز انعدام الثقة كعامل خطر في الاستعداد للأوبئة، وتخطيط الاستجابة لها. نحن نقترح مجموعتين من الاستراتيجيات القائمة على الأدلة: (1) استراتيجيات لضمان أن الثقة الموجودة بالفعل في المجتمع أثناء الأزمة، تستمر أثناء الأزمة، مثل تخفيف الإرهاق الناجم عن الجائحة بواسطة التدخلات الصحية والتواصل الصادق والشفاف والنزيه؛ (2) استراتيجيات لتعزيز التعاون في المجتمعات التي لا يثق فيها الناس بحكوماتهم وجيرانهم، لأسباب تاريخية مشروعة في بعض الأحيان. وحيثما توجد حالة من انعدام الثقة، فإن الاستعداد للوباء والاستجابات له يجب أن يعتمد بشكل أقل على الإكراه، وأكثر على تصميم السياسات المحلية، وبناء الشراكات مع مؤسسات المجتمع وقادته لمساعدة الناس على التغلب على الصعوبات التي يواجهونها في التعاون مع توجيهات الصحة العامة. إن المراقبة الدورية للثقة بين الأشخاص، والثقة في الحكومة على المستويين الوطني والمحلي، هي وسيلة لتمكين هذا الاستعداد للوباء والتخطيط للاستجابة له في سياق محدد.

## 摘要

尽管人们普遍承认在大流行病蔓延时信任非常重要，但是极少有人就如何将建立信任纳入下一场健康危机防范工作范畴提出具体的建议。其中一个原因是人们理所当然地认为建立信任是一个非常缓慢且具有挑战性的过程。尽管对公共机构的信任和互相信任对于防范大流行病至关重要，但是各国也应做好培养或恢复信任的工作有可能会付诸东流的准备。将建立信任纳入大流行病防范工作的范畴意味着承认两极分化、党派偏见和错误信息可能会持续存在，且我们需基于社区当前状态而非我们所希望其呈现的状态与其进行互动。本文提出了一项实用的政策议程，将不信任作为一项风险因素纳入大流行病防范和应对工作的规划。我们提出了两套循证策略：（i）确保在危机期间继续维持社区中已经建立的信任关系的策略，例如通过卫生干预措施和秉持真诚透明原则开展的有意义沟通来缓解流行病疲劳；以及（ii）在人们不信任政府及其邻居（有时因合理的历史原因）的社区实施旨在促进合作的策略。如果发现不信任的情况，则在制定大流行病防范和应对措施时，必须减少实施高压政策，同时依据当地实际情况制定当地政策并与社区机构和领导人建立伙伴关系，从而帮助人们克服困难以配合实施公共卫生指南。定期监测国家和地方层面的人际和政府信任情况，有利于制定符合具体实情的大流行病防范和应对工作规划。

## Резюме

Несмотря на широкое признание того, что доверие играет важную роль во время пандемии, существует мало конкретных предложений о повышении уровня доверия при подготовке к следующему кризису в области здравоохранения. Одна из причин заключается в том, что укрепление доверия справедливо воспринимается как медленный и сложный процесс. Хотя доверие к государственным учреждениям и друг к другу имеет большое значение для подготовки к пандемии, странам следует учитывать возможность того, что усилия по формированию или восстановлению доверия могут оказаться безуспешными. Включение доверия в процесс обеспечения готовности к пандемии означает признание факта сохранения поляризации, предвзятости и дезинформации, а также взаимодействие с сообществами в их текущем состоянии, а не в том, в котором их хотелось бы видеть. В данной работе представлена практическая программа действий по учету недоверия как фактора риска при планировании готовности к пандемии и ответных мер. В данной статье предлагаются два набора стратегий, основанных на фактических данных: (i) стратегии поддержания доверия, уже существующего в сообществе, во время кризиса, например смягчение пандемической усталости путем оказания медицинской помощи и честной и прозрачной коммуникации; (ii) стратегии развития сотрудничества в сообществах, где люди не доверяют своим правительствам и соседям, иногда по вполне обоснованным, историческим причинам. Там, где существует недоверие, меры по обеспечению готовности к пандемии и ответные меры должны в меньшей степени основываться на принуждении, а в большей – на разработке локальной политики и налаживании партнерских отношений с общественными институтами и лидерами с целью помочь людям преодолеть трудности, с которыми они сталкиваются при сотрудничестве с органами здравоохранения. Регулярный мониторинг межличностного и правительственного доверия на национальном и местном уровнях является одним из способов планирования готовности к пандемии и ответных мер с учетом конкретных условий.

## Introduction

Many studies of how well countries responded to health crises acknowledge the importance of trust.^1^^–^^5^ Higher levels of trust in government and interpersonal trust (i.e. trust in others) early in the coronavirus disease 2019 (COVID-19) pandemic were found to be associated with greater compliance with stay-at-home measures in studies of 19 European nations, and with greater hand-washing and mask-wearing in a study of 18 African Union Member States.^6^^–^^8^ After vaccines became available, trust in national health authorities and interpersonal trust were linked to higher vaccination coverage rates internationally and to greater willingness to heed health advice on the use of vaccines.^9^ With 4 years of the pandemic now behind us, trust in government and trust in others, as assessed in leading surveys, proved the best explanations for the substantial and persistent cross-country differences in COVID-19 outcomes in comparisons of similar countries that adjusted for relevant biological factors, such as age and the prevalence of key pre-existing comorbidities.^10^ Similarly, the results of a large study of inter-state differences in the United States of America highlighted the role of interpersonal trust.^11^

This research on the role of trust in responses to health emergencies suggests that the most effective way for a government to protect its citizens during a crisis is to persuade them to take voluntary measures to protect themselves and one another. However, implementing protective measures (e.g. contact tracing, social distancing, isolation, mask-wearing and vaccination) in a crisis involves changes in personal and community behaviour, which most governments find hard to monitor or compel at a population level. Accordingly, widespread adoption of protective measures, even when mandated, depends on the public’s cooperation. Several studies have shown that cooperation depends on trust between citizens and their governments and among citizens themselves, especially in democracies.^12^^,^^13^ Individuals are more likely to cooperate with recommended or mandated measures when they perceive their governments as trustworthy (i.e. that the government knows what it is doing and that it is acting for the common good) and they believe that public health programmes will be administered fairly and competently.^14^^,^^15^ Having greater trust in other people helps motivate individuals to cooperate to protect others in the community. Trust also reduces their fear of being misled into adopting protective measures when almost no one else is doing so.^11^^,^^16^^,^^17^

With the number of dangerous disease outbreaks on the rise globally, the ability to mobilize the public’s trust in a health crisis has never been more essential – or harder to maintain. Growing political polarization, persistent racial and social inequities, and rapidly changing medical technology may make it easier than ever for minor medical concerns and misinformation to erode public confidence.^18^ In particular, the rise of social media and artificial intelligence has made it simpler and cheaper to spread and amplify false information and to disseminate fake news stories than was previously possible with traditional media.^19^^,^^20^ Under the fast-changing circumstances of a pandemic, uncertainty, fear and anxiety can help the purveyors of mis- and disinformation to undermine the public’s trust that science and health systems can provide them with the best medical countermeasures.^21^

Despite widespread acknowledgement of the importance of trust during a pandemic, few intergovernmental institutions or governments have formulated concrete proposals on how to monitor trust in government and interpersonal trust, or on how to incorporate such assessments into preparing for the next health crisis.^22^^–^^24^ One reason is that, although research on the importance of trust in pandemic responses “makes intuitive sense”, as Bill Gates argued in his 2022 book on preparing for the next pandemic,^25^ such research does not “easily translate into practical advice” to donors and policy-makers because “building trust between people and their government takes years of painstaking, purposeful work”.^15^^,^^25^ Building social trust – the belief that most people can be trusted – is no easier. Some scholars suggest that progress on social trust requires systemic shifts, such as reducing corruption and economic inequality, and maintaining fair and efficient state institutions.^26^^,^^27^ Another concern is that much of the research on trust to date has taken place in Europe and North America. Consequently, this research may not be generalizable to other settings. Nevertheless, there is enough emerging evidence from low- and middle-income countries to raise concerns about whether governments are paying sufficient attention to the role of trust in planning effective pandemic responses.^4^^,^^5^^,^^7^^,^^28^

Although building faith in public institutions and one another is essential in preparing and responding to pandemics, countries should plan for the possibility that efforts to instil or restore that faith may fail. To that end, governments should prepare to engage with communities as they currently are, not as we would wish them to be. Pandemic preparedness should involve identifying and listening to communities’ reasons for mistrust in institutions and others, and monitoring and managing that mistrust as a pandemic risk.^29^ Consequently, governments should use the lessons learnt from monitoring mistrust to develop local pandemic plans that guard against the deterioration of trust during an emergency, and that can succeed even in communities where the level of trust is low.

Here we present a practical policy agenda for incorporating mistrust as a risk factor into pandemic preparedness and response planning. Our proposal includes two sets of concrete, evidence-based strategies: (i) strategies for ensuring the trust that already exists in a community is sustained during a crisis; and (ii) strategies for engaging and promoting cooperation even in communities where distrust runs deepest. Our approach to developing these proposals is detailed in Box 1.

Box 1 **Development of policy proposals for incorporating trust into pandemic preparedness and response, 2024**

In developing our policy proposals for incorporating trust into pandemic preparedness planning, we: (i) conducted thorough literature reviews of the Scopus, Google Scholar and PubMed® databases in April 2023 and January 2024 by searching for, for example, the terms trust, compliance and cooperation in article abstracts or titles, without any language or date restrictions; (ii) incorporated lessons from our personal research and activities in the coronavirus disease 2019 (COVID-19) pandemic, during which one of us served as an advisor to the Danish government on a national trust-monitoring and social-listening project; and (iii) took into account feedback from a consultation on a preliminary version of this proposal organized by WHO’s Health Emergencies Programme on 25 May 2023, which involved a wide range of representatives from WHO Member States, researchers, experts and members of civil society.^30^^,^^31^

WHO: World Health Organization.

## Sustaining trust during a health emergency

The degree of trust present in any society has specific historical, cultural and political roots.^26^^,^^27^ Trust is often built slowly over decades rather than months but can disappear quickly. A key concern for any decision-maker or authority during a health emergency is to not lose the trust already in place. This section highlights insights from the COVID-19 pandemic and previous epidemics on how to sustain trust during a crisis.

### Buffer pandemic fatigue

Pandemic fatigue demotivates compliance with health interventions and erodes trust in government guidance and crisis management generally.^32^ The concept of pandemic fatigue emerged during the COVID-19 pandemic and refers to the accumulated exhaustion that occurs over a prolonged crisis due to: (i) the emotional, psychosocial and material costs of complying with extended public health and social measures; and (ii) a constantly shifting information landscape concerning the threat and utility of those public health measures.^32^ As these feelings accumulate over time, interventions imposed later in a crisis or sustained longer may fuel more fatigue than interventions imposed during its early days.^32^ Pandemic fatigue may also spur individuals to consume more alternative media and misinformation, which could further undermine trust more broadly in government guidance and interventions. In the most severely affected individuals, this fatigue may be one source of radicalization and violent protest during a health emergency.^33^ Research suggests that the more severe the epidemic, the less the level of fatigue – all else being equal.^32^ People can handle high compliance costs if complying feels meaningful and there is a clearly defined exit strategy.

Policy-makers can mitigate pandemic fatigue using health interventions in two ways. First, decision-makers can reduce the unnecessary cost and inequity of emergency health interventions as much as possible.^32^ Policy-makers should: (i) invest in social protection policies and programmes to buffer the economic strain that accompanies stay-at-home orders, restrictions on gatherings, and other emergency measures; (ii) identify groups with psychosocial vulnerabilities and provide access to support; and (iii) scale back public health measures as soon as the epidemiological situation allows. Second, interventions need to make sense to the public and be perceived as meaningful. This approach requires being transparent about the current evidence base for interventions. Entering into dialogue with local communities on societal trade-offs can help policy-makers better align emergency health interventions with local cultural values and economic and educational needs.

### Invest in sense-making communication

Knowledge, evidence and recommendations can change rapidly during an emergency. Consequently, crisis communicators face a core dilemma: it is often not possible to provide a straightforward message that is consistent over time, yet communication needs to be timely and consistently actionable. Here, we refer to communication that navigates this dilemma as sense-making communication because it enables the public to make sense of health advice and understand why that advice is likely to change over the course of an emergency.

During the COVID-19 pandemic, an individual’s feeling of efficacy was associated with increased compliance with health authorities’ advice and with greater support for pandemic management.^34^ People feel efficacious when they: (i) have a clear sense of what to do; (ii) understand why they need to comply with a measure to deal effectively with a threat; and (iii) can comply without too high a cost.^35^ Research during the pandemic indicated that feelings of efficacy were elicited by transparent and honest descriptions of the overall strategy for handling the epidemic and of the exact role of the individual in that strategy.^36^ A challenge, of course, is that the strategy and the role of the individual will shift over the course of the pandemic.

Transparency and honesty are at the core of sense-making communication. Research during the COVID-19 pandemic showed that acknowledging the negative side-effects of a vaccine could lower uptake but increased the public’s trust in the health communicator and raised trust generally.^37^ In contrast, providing vague assurances about a vaccine’s safety lowered both acceptance of the vaccine and trust in health communicators. These observations confirm earlier research which found that the transparent communication of uncertainty preserves trust in the communicator and trust overall.^38^

Although the goals of clarity and transparency may seem to be in tension, effective sense-making communication should nonetheless strive to achieve both. Sense-making communication should provide clear, actionable advice about recommended public behaviour and admit any relevant uncertainties. To earn the trust of the public, health communicators must in turn trust the public to manage complex, concerning and changing information.^39^^,^^40^

Although research shows transparency has a positive effect in both high- and low-trust settings, strategies involving transparency may be more successful in societies that already have a high level of trust.^37^ Partisans and opportunists may exploit revelations of uncertainty to sow doubt in ethnically or politically polarized settings. During the COVID-19 pandemic, people in states and counties in the United States that voted heavily for the Republican presidential candidate in 2020, or that had a higher level of consumption of conservative news sources, stayed at home less, used masks less and had lower vaccine coverage.^11^^,^^41^

Given the fear that transparent communication may backfire in low-trust environments, future health crisis communicators should tailor their engagement with communities to local needs by liaising with community groups and leaders. This topic is explored in greater depth in the section on fostering cooperation in communities with low levels of trust in government and their neighbours.

The challenge of providing sense-making communication in a crisis becomes more difficult if that crisis involves an infodemic, which the World Health Organization (WHO) has defined as occurring when “too much information including false or misleading information in digital and physical environments” proliferates during a disease outbreak.^42^ During the COVID-19 pandemic, people were highly active in seeking information. Even though they overwhelmingly turned towards trustworthy information sources, the sheer amount of information complicated the task of health communicators.^43^

In a crisis, extreme and critical voices may join together to create online echo chambers, which are usually driven by a small group of highly active individuals.^44^ During the COVID-19 pandemic, up to 65% of vaccine-related misinformation on mainstream social media was attributable to just 12 highly active accounts.^45^ Research into understanding how authorities can engage constructively with activist online groups is still in its infancy. Key components are likely to be: (i) a strong online presence for health authorities; (ii) investment in digital literacy to promote resilience against mis- and dis-information; and (iii) cooperation with social media platforms to ensure their algorithms do not amplify harmful disinformation during an emergency.

### Avoid sowing social tension

An effective response to a public health emergency requires collective action whereby individuals limit the exercise of their individual freedoms to advance the interest of the group, for example by changing their behaviour to suppress the transmission of dangerous pathogens to protect the most vulnerable.^46^ Such collective action requires social trust – the trust people have that other people are also contributing to the joint action.^47^ A standard finding in psychological and economic research is that people react with anger and limit their own contributions when those not contributing are observed to free-ride on others’ joint efforts.^48^

One way in which mistrust between citizens emerged during the COVID-19 pandemic was via the moral condemnation of people who did not observe the advice of health authorities.^49^ Across both developed and developing countries, individuals who were vaccinated against COVID-19 were more likely to exclude unvaccinated individuals from family interactions.^50^ In addition, decision-makers used moral rhetoric to justify interventions that were particularly burdensome to those who were not vaccinated.^51^ Such feelings of antipathy also led to measures – and public support for measures – that arguably restricted freedom of movement and speech.^50^^–^^53^ In turn, individuals who were not vaccinated felt pressured and discriminated against, with the result that groups whose trust in the authorities and in pandemic responses was already limited became even less trusting.^54^^,^^55^

Research shows that condemnation is common in the context of collective action,^56^ but it was often counterproductive during the COVID-19 pandemic. Health authorities should invest in building a thorough and nuanced understanding of the factors underlying opposition to their advice. This understanding could improve responses to that opposition and help authorities communicate with the rest of the public about those concerns. For example, during the COVID-19 pandemic, an unvaccinated person may have had: (i) an underlying medical condition; (ii) negative past experiences with the health authorities (e.g. as a member of an ethnic or racial minority); (iii) safety concerns due to prior public health scandals; or (iv) ethical questions about vaccine equity. Reducing such a complex mix of considerations to a moral position or a simplified stereotype can entrench opposition in the subset of unvaccinated people who could still be persuaded to adopt public health measures.

## Fostering cooperation in communities with limited trust

Governments need pandemic strategies that can succeed in the communities that currently exist, not just in the cohesive communities they hope to build. Incorporating trust into pandemic preparedness involves: (i) acknowledging that polarization, partisanship and misinformation may persist; (ii) monitoring trust at national and subnational levels; and (iii) engaging local institutions and community leaders to develop strategies to foster cooperation even in communities with limited trust in either government or their neighbours.

Although relatively few studies have examined cooperation in societies with low levels of trust, there has been research in regions where people have legitimate, historical reasons to mistrust their governments and neighbours.^57^^,^^58^ The first lesson this research teaches is that mandates can generate instability, pushback and conflict in divided communities. Although a mandate can spur cooperation in more harmonious societies by convincing potential holdouts that everyone else will comply, research suggests they may have the opposite effect in low-trust communities.^59^ A study of 41 communities with low levels of government trust during the COVID-19 pandemic found no link between stringent rules and greater cooperation with hand-washing or physical distancing.^60^ Especially in such settings, governments must rely less on coercion and more on building policies that can help people overcome the difficulties they encounter in cooperating with public health guidance. For example, states in the United States that employed social protection policies, such as paid family and sick leave, appeared to have increased compliance with emergency response measures among people who otherwise could not afford to comply.^29^

The second lesson that emerges from this research is that cultural, religious and kinship ties can help low-trust communities set aside historical mistrust and suspicions about government measures. During the 2014 Ebola virus disease outbreak in Liberia, for instance, viral transmission rates started falling after governments and nongovernmental organizations recruited community youth leaders, pastors and imams to check households for infected people. Likewise, in Sierra Leone, community liaisons helped increase acceptance of Ebola vaccine trials.^61^ In the United States, local churches, community vaccine ambassadors and even local barber shops provided focal points encouraging cooperation around COVID-19 vaccination in majority Black communities with historical reasons to distrust public health campaigns.^62^ Even in settings where management of the COVID-19 pandemic was politicized, endorsement of vaccines by trusted community leaders increased acceptance among community members.^63^

Ultimately, cultural, political and religious ties that can spread misinformation can also foster the spread of accurate information and motivate cooperation with effective public health measures.^18^ Partnering with local physicians, health centres and faith-based institutions can help identify trusted messengers, improve data collection and advance public health priorities.^64^ Moreover, community engagement in planning and decision-making may help to ensure local values and preferences are respected and are used to guide communication with the community during future crises.^65^

## Monitoring trust during a health emergency

Investing in the real-time monitoring of the public’s trust in decision-makers, scientists, health authorities, the health system and each other is necessary to assess levels of community trust levels and the impact of health interventions on trust. When appropriate, authorities should also monitor the trust in the overall emergency response strategy.

The infrastructure for monitoring trust should be set up in advance of a crisis to assess the baseline level of trust in a community, and it should incorporate standardized measures of trust from the burgeoning research literature. At the country level, data on some baseline measures are already available through existing sources, such as the World Values Survey.^66^ Such large-scale surveys, however, do not provide data at the subnational level for individual countries.

As part of a pandemic preparedness and response plan, a dedicated trust monitoring unit could be established by, or in partnership with, university researchers with the relevant social science expertise. The appropriate infrastructure for trust monitoring would involve: (i) a system for collecting fine-grained, valid data on trust; (ii) a system for processing, analysing and interpreting these data in real time to guide responses to adverse events; and (iii) a mechanism for conveying information to relevant policy-makers and decision-makers at subnational and national levels. Extensive subgroup analyses must also be conducted to identify communities with particularly low levels of trust to guide the design and deployment of suitable interventions. Moreover, the data produced should be openly available to build and sustain confidence in efforts to monitor trust.

For any assessment of the public’s level of trust to be useful, the data collected must be representative. However, members of groups that do not trust the government are more likely to opt out of surveys and, in addition, other research approaches (e.g. social media monitoring) may not adequately capture the sentiments of the silent majority. A lack of adequate representativeness can result in false estimates of the level of trust and the misidentification of factors influencing trust.^67^

Accordingly, a successful strategy for monitoring mistrust as a risk to an effective health emergency response must rely on a combination of methods, such as representative online surveys, social media monitoring and ethnographic field observations. Surveys can assess the public’s trust that the government and other members of the community will respond effectively to a health emergency. Social media monitoring will provide information about the views of the most vocal individuals and about misinformation that is circulating, whereas ethnographic field observations can collect in-depth information on considerations important to different groups and communities.^68^ Use of this combination of methods is consistent with the concept of social listening within infodemic management that WHO proposed during the COVID-19 pandemic. Infodemic management is intended to be a practical means of identifying and addressing the questions, concerns, information voids, perceptions, behaviours, and mis- and disinformation that may be spreading through communities during a health emergency.^42^^,^^69^ A concrete example of the feasibility of large-scale social listening was the HOPE project (i.e. how democracies cope with COVID-19) in Denmark,^70^ which used all three methods of social listening mentioned here.

In essence, a trust monitoring system is a dedicated system for carefully listening to popular concerns about government and community responses to a health crisis, and for integrating those concerns into the decision-making process guiding those responses. Although it requires time, money and training to succeed, trust monitoring did prove effective during the COVID-19 pandemic.^71^ That said, there is much more to learn about the role of trust in pandemic preparedness. This statement is especially true in low- and middle-income countries, where researchers are developing locally tailored, behavioural science approaches that can contribute to global understanding of the role of trust in health emergencies.^72^

## Conclusion

An effective pandemic response requires the cooperation of billions. The COVID-19 pandemic revealed that many societies around the globe were too divided and riven by mistrust to mobilize their citizens to protect themselves and others during a crisis. Although restoring faith in public health institutions and one another is essential, countries must prepare for failure by developing strategies that encourage cooperation in communities as they currently are. Low public trust is a pandemic risk factor that should be monitored and mitigated to enable public health and social responses to health emergencies to succeed, even in communities with a historical mistrust of government and their neighbours. Being alert to changes in public confidence, nurturing cooperation amid polarization and social strife, and tailoring nimble policy responses to local needs will all be critical for ensuring better outcomes when the next dangerous outbreak occurs.
